# Hydrogen-Rich Water as a Novel Therapeutic Strategy for the Affective Disorders Linked with Chronic Neuropathic Pain in Mice

**DOI:** 10.3390/antiox11091826

**Published:** 2022-09-16

**Authors:** Maria Martínez-Serrat, Ignacio Martínez-Martel, Santiago Coral-Pérez, Xue Bai, Gerard Batallé, Olga Pol

**Affiliations:** 1 Grup de Neurofarmacologia Molecular, Institut d’Investigació Biomèdica Sant Pau (IIB SANT PAU), Sant Quintí 77-79, 08041 Barcelona, Spain; 2 Grup de Neurofarmacologia Molecular, Institut de Neurociències, Universitat Autònoma de Barcelona, 08193 Barcelona, Spain

**Keywords:** analgesia, apoptosis, hydrogen-rich water, inflammation, molecular hydrogen, neuropathic pain, oxidative stress

## Abstract

Neuropathic pain manifested with allodynia and hyperalgesia usually becomes a chronic condition accompanied with mood disorders. Clinical therapies for neuropathic pain are still unsatisfactory with notable side effects. Recent studies have reported the protective role of molecular hydrogen (H_2_) in different diseases including neurological disorders, such as Alzheimer’s as well as its antidepressant activities in animals with chronic stress. This study explored the effects of treatment with hydrogen-rich water (HRW) in male mice with neuropathic pain induced by the chronic constriction of the sciatic nerve (CCI) and the accompanying affective deficits. The likely pathways implied in the HRW analgesic activity, as well as the interaction between heme oxygenase 1 (HO-1) enzyme and H_2_ during neuropathic pain were also studied. The results showed: (i) the inhibitory effects of the repetitive treatment with HRW on the allodynia and hyperalgesia provoked by CCI; (ii) the anxiolytic and antidepressant actions of HRW in animals with neuropathic pain; (iii) the contribution of the antioxidant enzymes (HO-1 and NAD(P)H: quinone oxidoreductase 1) and the ATP sensitive potassium channels in the painkiller activities of HRW during neuropathic pain; (iv) a positive interaction between the HO-1 and H_2_ systems in inhibiting the CCI-induced neuropathy; and (v) the antioxidant, antinociceptive, anti-inflammatory and/or antiapoptotic features of HRW treatment in the dorsal root ganglia and/or amygdala of sciatic nerve-injured mice. This study demonstrates new protective actions of H_2_ and suggests that treatment with HRW might be an interesting therapeutic strategy for chronic neuropathic pain and its associated mood disorders.

## 1. Introduction

Neuropathic pain caused by a lesion or disease directly affects to the peripheral (PNS) or central nervous system (CNS). Some examples of conditions that can cause a neuropathy are the spinal cord or sciatic nerve injury, chemotherapy, and diabetes, among others [1]. This condition is known to become chronic and has a big negative impact on the patients’ quality of life with a prevalence of 7–10% in the general population.

The main symptoms of chronic neuropathic pain (CNP) are burning, sharp electric-like sensations, allodynia, and hyperalgesia [2]. Numerous comorbidities have been also widely observed in patients enduring CNP, the most frequent are related with the emotional disorders such as anxiety and depression [3,4]. Nowadays, there is a wide variety of recommended first-line treatments for CNP targeting different pathways for instance, tricyclic antidepressants, anticonvulsants, or serotonin and noradrenaline reuptake inhibitors. However, all these approaches have been proved to be insufficient and with significant secondary effects [5,6]. As a result, the allodynia is only partially diminished and thermal hyperalgesia is usually still persistent with some of these treatments [1,2]. More remarkable is the point, that most of the current therapies do not inhibit or only reduce part of the emotional disorders linked with CNP. Therefore, it is important to find new treatments that successfully can alleviate pain and its related mood disorders.

The exact mechanisms concerned in the generation and maintenance of CNP are still not fully resolved, although inflammation and oxidative stress are two processes widely implicated [1]. An increase of reactive oxygen species (ROS) induces different physiological responses for example cell death and inflammation. It is known that during CNP, oxidative stress leads to cell death through an upregulation of Bcl2-associated X (BAX) in different brain areas [7]. As for the inflammatory responses, the overexpression of phosphoinositide 3-kinase (PI3K)/phosphorylated protein kinase B (p-Akt) and the nuclear factor-κB (NF-κB) transcription factor in both PNS and CNS, has been proved that contribute to pain sensitization [8,9,10].

The major endogenous antioxidant system is initiated by the translocation of the nuclear erythroid factor 2-related factor 2 (Nrf2) from the cytoplasm to the nucleus and the subsequent transcription of several antioxidant proteins, including heme oxygenase 1 (HO-1), superoxide dismutase 1 (SOD-1) and NAD(P)H: quinone oxidoreductase 1 (NQO1). The upregulation of these enzymes triggered by Nrf2 has exhibited a positive relationship with antinociceptive and anti-inflammatory responses in several pain models [11,12], as for example that induced by cobalt protoporphyrin IX (CoPP), an HO-1 inductor in animals with inflammatory and osteoarthritic pain [13,14]. The enzyme HO-1 catalysis the heme group into biliverdin, iron and carbon monoxide (CO) which acts as a gaseous neurotransmitter with antiapoptotic, antiproliferative and anti-inflammatory properties [12,15]. This gas activates different cascades, one of which is the cyclic 3′,5′-guanosine monophosphate/protein kinase G/ATP-sensitive potassium (K_ATP_) channels that produces analgesia [16].

Previous studies reported the interaction between the HO-1/CO pathway with other gaseous compounds in several rodent pain models. Thereby, the outstanding effects of hydrogen sulfide (H_2_S) in treating CNP through the administration of slow-releasing H_2_S donors, are mainly produced by activating the endogenous HO-1 expression [17,18]. A close interaction between H_2_S and CO as well as among nitric oxide and the HO-1/CO system in modulating chronic osteoarthritis and inflammatory pain in rodents has been also demonstrated [14,19]. Consequently, the combination of different gases constitutes a potential therapeutic target for chronic pain.

In the recent years, molecular hydrogen (H_2_) has been studied in a wide range of diseases because of its protective properties and the absence of demonstrable relevant secondary effects induced by this molecule [20]. The four main mechanisms through which H_2_ exerts its neuroprotective effects include the inhibition of oxidative stress, the inflammatory and apoptotic processes, as well as the regulation of autophagy [20,21]. H_2_ produces its antioxidant properties by neutralizing OH and ONOO radicals. This gas also improves the expression of antioxidant enzymes SOD-1 and HO-1. Treatment with H_2_ has also been linked with the inhibition of microglial activation and the secretion of pro-inflammatory cytokines (IL-1β, IL-6, TNF-α), together with the blockage of NF-κB pathway [20]. As for its anti-apoptotic properties, it has been observed an increased apoptosis regulator Bcl-2/BAX ratio and a decreased expression of p-Akt and glycogen synthase kinase-3β. Finally, some studies further suggested that H_2_ can regulate autophagy by decreasing the number of autophagosomes and altering the expression of different proteins for instance reducing extracellular signal-regulated kinase phosphorylation [20,22,23].

Therefore, the growing evidence on the therapeutic effects of H_2_, together with its capacity to cross the blood brain barrier and its limited side effects, makes this gas a very interesting new approach to treat several neurological diseases such as CNP. In consequence, previous studies have demonstrated the antinociceptive properties of the prophylactic treatment with hydrogen-rich water (HRW) or saline (HRS) in rodents with neuropathic pain induced by nerve injury [11,24,25,26] or spinal cord ligation [27,28]. Moreover, the antidepressant effects of HRW were also described in animals with mild chronic unpredictable stress [29]. Nevertheless, the possible analgesic actions of the therapeutic administration of HRW in mice with neuropathic pain provoked by the chronic constriction of the sciatic nerve (CCI) and its effects on the anxiety- and depressive-like behaviors associated have not been previously reported.

In male mice with CCI-induced neuropathic pain, our objectives are to assess: (i) whether the intraperitoneal therapeutic administration of HRW can alleviate the mechanical allodynia, thermal hyperalgesia, and cold allodynia generated by CCI; (ii) if the treatment with HRW can reverse the anxiety- and depressive-like behaviors linked with CNP; (iii) the possible pathways involved in the analgesic activities of HRW; (iv) the analgesic effects produced by the co-administration of CoPP with HRW; (v) the action of HRW treatment on the inflammatory, nociceptive, apoptotic and/or oxidative changes incited by CCI in the amygdala (AMG) and dorsal root ganglia (DRG).

## 2. Materials and Method

### 2.1. Animals

The animals used in this project were male C57BL/6 mice acquired from Envigo Laboratories (Barcelona, Spain). All mice were 6–8 weeks old and weighted between 21 and 26 g. They were housed at a standard 12/12 h light/dark cycle at a room temperature of 22 °C and humidity of 66% with free access to food and water. The rodents were accommodated for 7 days to the housing conditions before starting the experiments. All experiments were performed between 9:00 a.m. to 5:00 p.m.

This project was executed following the regulations of the European Commission’s directive (2010/63/EC), the Spanish Law (RD 53/2013) regulating animal research and approved by the Committee of Animal Use and Care of the Autonomous University of Barcelona (9863). Maximal efforts were made to achieve a reduced number of employed animals and their suffering. All experiments were accomplished by investigators blinded to the experimental circumstances.

### 2.2. Induction of Neuropathic Pain

The animals underwent the total ligation of sciatic nerve to induce CNP. The surgery was performed under isoflurane anesthesia (3% induction, 2.5% maintenance). By means of blunt dissection, the biceps femoris and gluteus superficialis were segmented to reach the sciatic nerve. Afterwards, three ligatures (4/0 silk) were attached around the sciatic nerve making sure the epineural circulation was preserved. As for the control groups, the same procedure was performed excepting nerve ligation (sham-operated).

### 2.3. Mechanical Allodynia

To determine the mechanical allodynia, animals were placed in Plexiglas tubes (20 cm high × 9 cm diameter) on a wire grid bottom. The hind paw withdrawal was measured by evaluating the response after the stimulation with different von Frey filaments ranging bending forces from 0.4–3.5 g (North Coast Medical, Inc., San Jose, CA, USA). The filaments were applied through the grid bottom using the up-down paradigm as previously reported [30]. The test begin with a filament of 0.4 g and the strength of the next filament was enhanced or diminished given that mice responses. Finally, the threshold of the answer was calculated from the sequence of responses using an Excel program (Microsoft Iberia, SRL, Barcelona, Spain) that incorporates the curve fitting of the data.

### 2.4. Thermal Hyperalgesia

Thermal hyperalgesia was measured using the plantar test (Ugo Basile, Varese, Italy), where the paw withdrawal latency was determined in reaction to radiant heat. For this test, mice were set in Plexiglas cylinders (20 cm high × 9 cm diameter) on a glass surface. The heat source located under the plantar surface of the hind paws was activated with a light beam intensity until the paw withdrawal. A cut-off time of 12 s was used to prevent paw damage. Mean paw-withdrawal latencies were determined from the average of three separate trials.

### 2.5. Cold Allodynia

Cold plate device (Ugo Basile, Varese, Italy) was used to assess cold allodynia. Mice were placed on a cold plate (4 ± 0.5 °C) during 5 min to determine the number of elevations of each hind paw.

All tests were realized on the ipsilateral and contralateral paws.

### 2.6. Anxiety-like Behaviors

The anxiety-like behaviors were studied through the elevated plus maze (EPM), an X-shaped structure with 2 open and 2 closed arms placed at 45 cm from the floor. All arms were 5 cm wide and 35 cm long and the closed ones had 15 cm high walls. At starting the test, the mouse was placed at the central area of the maze facing always the same open arm and its behavior was recorded for 5 min using a digital camera. The number of entries into the open and closed arms and percentage of time passed in the open arms were determined.

### 2.7. Depressive-like Behaviors

The depressive-like behaviors were assessed employing the tail suspension test (TST) and the forced swimming test (FST), where the time passed by the animal completely still was quantified as immobility time (s).

In the TST, animals were suspended by their tail using adhesive tape attached at 1 cm from the tip of the tail to a firm structure located at 35 cm above the floor and were recorded by a digital camera for 8 min. The immobility time was quantified during the last 6 min.

In the FST, mice were individually placed in a Plexiglass tube (25 cm high × 10 cm diameter) filled with water (24 °C ± 1 °C) at a height of 10 cm. Mice were recorded for 6 min and the immobility time during the last 4 min was measured.

### 2.8. Drugs

HRW was prepared by using a hydrogen water generator from Hydrogen (Osmo-star Soriano S.L., Alicante, Spain), that uses electrolysis method to break down H_2_O into H_2_. HRW was intraperitoneally injected 1 h before testing at a concentration of 0.3 mM. Tin protoporphyrin IX (SnPP) and CoPP were acquired from Frontier Scientific (Livchem GmbH & Co., Frankfurt, Germany), and dicoumarol was purchased from Eurodiagnostico S.L. (Madrid, Spain). All of them were dissolved in dimethyl sulfoxide (DMSO, 1% solution in saline (0.4% NaCl). Glibenclamide purchased from Sigma–Aldrich (St. Louis, MO, USA) was dissolved in saline solution. SnPP, glibenclamide and dicoumarol were administered 30 min before testing at a dose of 10 mg/kg, and CoPP at 3 h prior to the tests at dose of 2.5 mg/kg according to earliest studies [13,31]. All drugs were prepared right before their use and were intraperitoneally injected in a final volume of 10 mL/kg. For each treatment, the respective control group received the corresponding vehicle (VEH).

### 2.9. Experimental Protocol

In the first procedure, sham-operated and CCI-mice were therapeutically administered with VEH, HRW 1T (1 time/day) or HRW 2T (2 times/day) during 7 consecutive days. Mechanical allodynia, thermal hyperalgesia and cold allodynia were tested before the injury (baseline), one day prior to the treatment (pre-test; day 21 post-surgery) and in each day of treatment (days 22–28 post-surgery).

To study the effects of HRW on the anxiety- and depressive-like behaviors associated with CNP other sets of sham-operated and CCI-mice were used. According to the results obtained in the first experiments, HRW 1T groups were treated for 7 days, at days 22–28 post-surgery, and the HRW 2T groups were treated for 4 days, at days 25–28 post-surgery. In both groups the evaluation of the anxiety- and depressive-like behaviors were performed at the last day of treatment (day 28 after the surgery).

To study the possible pathways linked in the antinociceptive effects of HRW, sham-operated and CCI-animals were administered with VEH or HRW combined with VEH, SnPP (HO-1 inhibitor), dicoumarol (NQO1 inhibitor) or glibenclamide (K_ATP_ channels blocker) [11,31,32,33] at 2T during 4 consecutive days (days 25–28 post-surgery) and the nociceptive responses were evaluated at each day of treatment.

The analgesic effects of the co-administration of CoPP with HRW were also assessed. Sham-operated and CCI-animals were administered with VEH, HRW or CoPP, alone and combined, at 2T from days 25 to 28 post-surgery. The nociceptive responses were assessed in each day of treatment.

Lastly, the effects induced by administration of HRW 2T during 4 consecutive days in the expression of HO-1, SOD-1, NQO1, phosphorylated-NF-κB inhibitor alpha (p-IKBα), p-Akt, BAX and 4-hydroxy-2-nonenal (4-HNE) in the AMG and DRG were evaluated by Western blot.

### 2.10. Western Blot Analysis

At day 28 after surgery, sham-operated and CCI-mice were euthanized by cervical dislocation, then AMG and DRG were collected and kept at −80 °C until used. Tissues were sonicated in lysis buffer RIPA buffer, 0.5% protease inhibitor cocktail and 1% phosphatase inhibitor cocktail (Sigma-Aldrich, St. Louis, MO, USA). A second sonication (10 sec) was performed after 1 h solubilization at 4 °C. The samples were centrifuged at 4 °C for 20 min at 700× *g* and their supernatants were collected and mixed (60 µg of total protein) with 4 × Laemmli loading buffer. These samples were loaded onto a 4% stacking/12% separating sodium dodecyl sulphate polyacrylamide gel, where the electrophoresis took place. Next, proteins were electrophoretically transferred (120 min) onto a polyvinylidene fluoride membrane and blocked with phosphate-buffered saline plus Tween 20 (PBST) or Tris-buffered saline plus Tween 20 (TBST) comprising 5% non-fat dry milk or 5% bovine serum albumin for 75 min. Afterwards, membranes were incubated overnight at 4 °C with rabbit primary antibodies anti p-Akt (1:200), Akt (1:200) and BAX (1:150) from Cell Signaling Technology (Danvers, MA, USA); HO-1 (1:250), p-IKBα (1:150), IKBα (1:150), 4-HNE (1:200) from Abcam (Cambridge, UK); NQO1 (1:250) from Sigma-Aldrich (St. Louis, MO, USA); SOD-1 (1:150) from Novus Biologic (Littleton, CO, USA); and anti-glyceraldehyde-3-phosphate dehydrogenase (GAPDH, 1:5000) from Merck (Billerica, MA, USA) as a loading control. Blots were incubated for 1 h at room temperature with an anti-rabbit secondary antibody conjugated to horseradish peroxidase (GE Healthcare, Little Chalfont, United Kingdom). Proteins were visualized by chemiluminescence adding ECL kit reagents (GE Healthcare, Little Chalfont, United Kingdom) and detecting the photon emission with Chemidoc MP imaging system (Bio Rad, Hercules, CA, USA). Finally, densitometric analysis was performed with the Image-J software (National Institutes of Health, Bethesda, MD, USA).

### 2.11. Statistical Analysis

Data are expressed as mean values ± standard error of the mean (SEM). The program used to do the statistical analysis was SPSS (version 28, IBM, Madrid, Spain). The performed analysis was a three-way repeated measures analysis of variance (ANOVA) to study differences in surgery, treatment and days of treatment and their possible interactions. For each day, a one-way ANOVA with a Bonferroni post hoc test was carried out to evaluate the effects of the different treatments on the mechanical allodynia, thermal hyperalgesia and cold allodynia induced by CCI.

To examine the effects of treatments in the anxiety- and depressive-like behaviors associated with CNP, a one-way ANOVA followed by a Bonferroni test was performed. To analyze the changes in the protein levels, a one-way ANOVA followed by a Bonferroni test was also used.

In all cases, *p* < 0.05 was considered significant.

## 3. Results

### 3.1. Treatment with HRW Inhibited the Nociceptive Symptoms Induced by CCI

**Figure 1:**
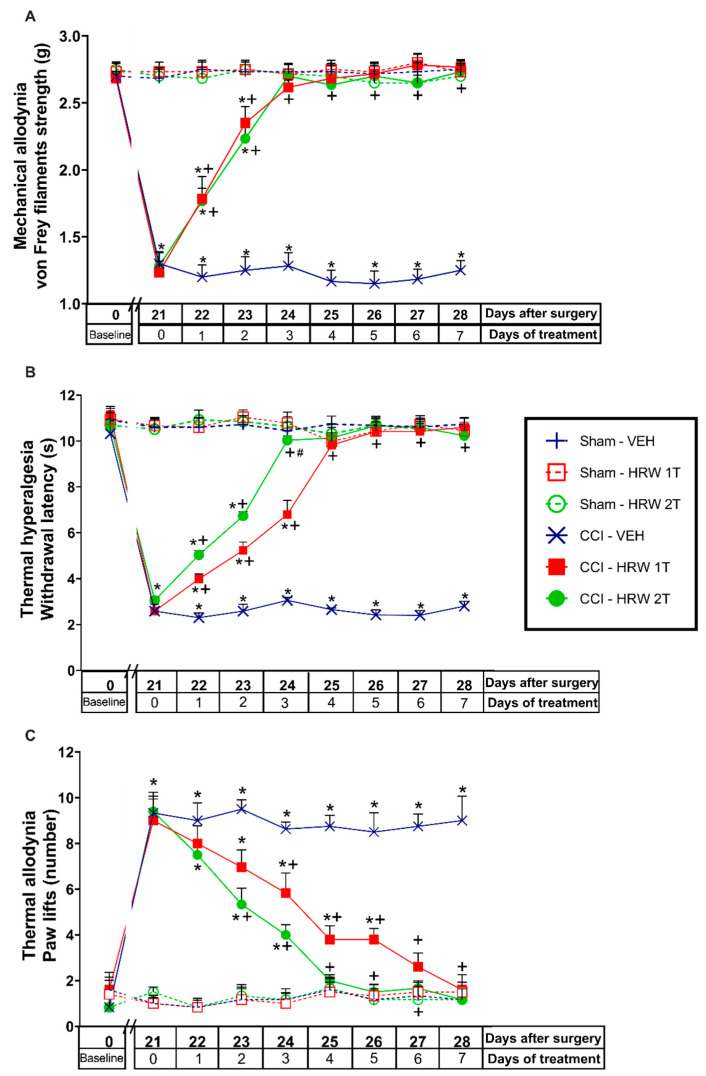
Effects of the repetitive administration of HRW at 1T or 2T in the allodynia and hyperalgesia induced by CCI. Mechanical antiallodynic (**A**), thermal antihyperalgesic (**B**), and thermal antiallodynic effects (**C**) produced by the repeated administration of HRW 1T or 2T from days 22 to 28 after surgery are represented. Sham-operated mice were used as controls. In all pictures, for each treatment and day evaluated, * indicates significant differences vs. sham-operated VEH-treated animals, + vs. CCI–VEH-treated animals and # vs. CCI–HRW 1T treated animals (*p* < 0.05, one-way ANOVA followed by the Bonferroni test). Mean values ± SEM (n = 6 animals per group).

In CCI-mice intraperitoneally injected with either VEH or HRW 1T or 2T during 7 consecutive days, their effects on the mechanical allodynia, thermal hyperalgesia and cold allodynia induced by nerve injury were evaluated. Sham-operated animals were used as controls. For the mechanical allodynia, the three-way repeated measures ANOVA showed significant effects of the surgery, treatment, and days of treatment (*p* < 0.001). Moreover, interactions between surgery and treatment, surgery and days of treatment, treatment, and days of treatment (*p* < 0.001), and among the three factors (*p* < 0.001) were also demonstrated. The results showed that the reduced threshold of the ipsilateral hind paw withdrawal to von Frey filaments stimulation induced by CCI at 21 days after surgery (*p* < 0.001, one-way ANOVA vs. sham-operated mice treated with VEH; Figure 1A) was completely reduced after 3 days of treatment with HRW injected at 1T or 2T.

For the thermal hyperalgesia, the three-way repeated measures ANOVA also showed significant effects of the surgery, treatment, and days of treatment (*p* < 0.001), and interactions between surgery and treatment, surgery and days of treatment, treatment and days of treatment (*p* < 0.001), and among the three factors (*p* < 0.001). Moreover, the reduced withdrawal threshold of the ipsilateral hind paw in response to a thermal stimulus produced by CCI at 21 days after surgery (*p* < 0.001, one-way ANOVA vs. sham-operated mice treated with VEH; Figure 1B), was also reduced by HRW, but while 3 days of treatment with HRW 2T completely blocked the thermal hyperalgesia, 4 days of treatment with HRW 1T were necessary.

Finally, for the cold allodynia, the three-way repeated measures ANOVA also showed significant actions of the surgery, treatment, and days of treatment (*p* < 0.001); and interactions between surgery and treatment, surgery and days of treatment, treatment, and days of treatment (*p* < 0.001), and between the three factors (*p* < 0.001). Thus, the enhanced number of ipsilateral hind paw lifts caused by cold stimulus at day 21 after surgery (*p* < 0.001, one-way ANOVA vs. sham-operated mice treated with VEH; Figure 1C) was reversed with 4 or 6 days of treatment with HRW 2T or 1T, respectively.

In all tests and for each evaluated time, no significant differences were observed between animals treated with HRW at 1T or 2T. The only exception was in the inhibition of thermal hyperalgesia (Figure 1B), where the effects induced by HRW 2T at the third day of treatment are higher than those produced by HRW 1T (*p* < 0.001, one-way ANOVA).

No alterations were found in any test or time evaluated in the contralateral paws of CCI or sham-operated animals treated with VEH or HRW at 1T or 2T (data not shown).

### 3.2. Treatment with HRW Inhibited the Anxiety and Depressive-like Behaviors Associated with CNP

To evaluate the impact of HRW treatment on the anxiety- and depressive-like behaviors associated with CNP, mice were treated with either VEH or HRW 1T or 2T during 7 or 4 days, respectively. At 28 days after surgery, the EPM test is used to evaluate the anxious-like behaviors and the TST and FST to study the depressive-like behaviors.

**Figure 2:**
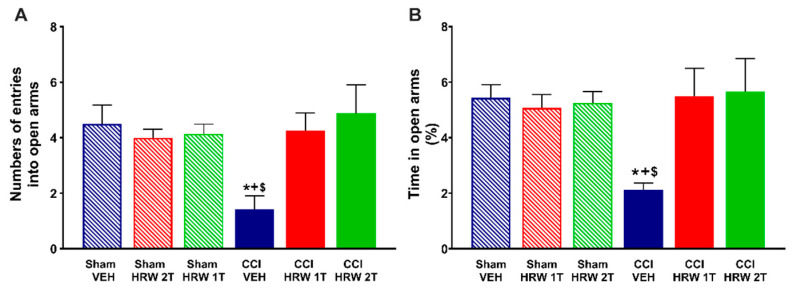
Treatment with HRW at 1T or 2T inhibited the anxiety-like behaviors associated with neuropathic pain. At day 28 after surgery, the number of entries into the open arms (**A**) and the percentage (%) of time spent in them (**B**) after 4 or 7 consecutive days of treatment with VEH, HRW 2T or 1T are represented. Sham-operated mice treated with VEH, HRW 2T or 1T are also displayed. For each test, * indicates significant differences vs. sham-operated mice treated with VEH or HRW at 1T or 2T, + vs. CCI-HRW 1T treated mice and $ vs. CCI-HRW 2T treated mice (*p* < 0.05, one-way ANOVA followed by Bonferroni test). Mean values ± SEM (n = 8 animals per group).

For the EPM, the one-way ANOVA showed a reduction in the number of entries into the open arms (*p* < 0.05, vs. sham-operated mice treated with VEH, Figure 2A) and in the percentage of time spent in them (*p* < 0.05, one-way ANOVA vs. sham-operated mice treated with VEH, Figure 2B) that were reversed by both treatments, HRW 1T administered for 7 days and HRW 2T administered for 4 days.

Possible abnormalities in the locomotor activity were evaluated by analyzing the number of entries in the closed arms. No changes were observed in any of the groups studied (data not shown).

**Figure 3:**
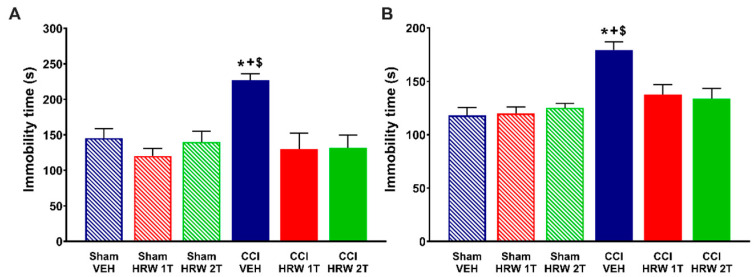
Treatment with HRW at 1T or 2T inhibited the depressive-like behaviors associated with neuropathic pain. The immobility time (s) was evaluated in the TST (**A**) and FST (**B**) at day 28 after surgery, after 4 or 7 consecutive days of treatment with VEH, HRW 2T or HRW 1T, respectively. Sham-operated mice treated with VEH, HRW 1T or HRW 2T are also displayed. For each test, * indicates significant differences vs. sham-operated mice treated with VEH or HRW at 1T or 2T, + vs. CCI-HRW 1T treated mice and $ vs. CCI-HRW 2T treated mice (*p* < 0.05, one-way ANOVA followed by Bonferroni test). Mean values ± SEM (n = 8 animals per group).

For the depressive-like behaviors, an increase in the immobility time in CCI mice treated with VEH was revealed in both tests, the TST (Figure 3A) and FST (Figure 3B) (*p* < 0.05, one-way ANOVA vs. sham-operated mice treated with VEH) at 28 days after surgery. In both tests, the enhanced immobility time was significantly inhibited after treatment with HRW 1T for 7 days, or HRW 2T for 4 days, and no significant differences were found between sham-operated mice treated with VEH, HRW at 1T or 2T.

### 3.3. Reversion of the Antinociceptive Effects of HRW with Specific HO-1 and NQO1 Inhibiters and a K_ATP_ Channels Blocker

Since the analgesic effectiveness induced by the administration of HRW 2T is higher than those produced by HRW 1T, the impact of SnPP (HO-1 inhibitor), dicoumarol (NQO1 inhibitor) or glibenclamide (K_ATP_ channels blocker) treatments in the HRW pain killer effects was assessed in animals treated with HRW 2T during 4 consecutive days.

**Figure 4:**
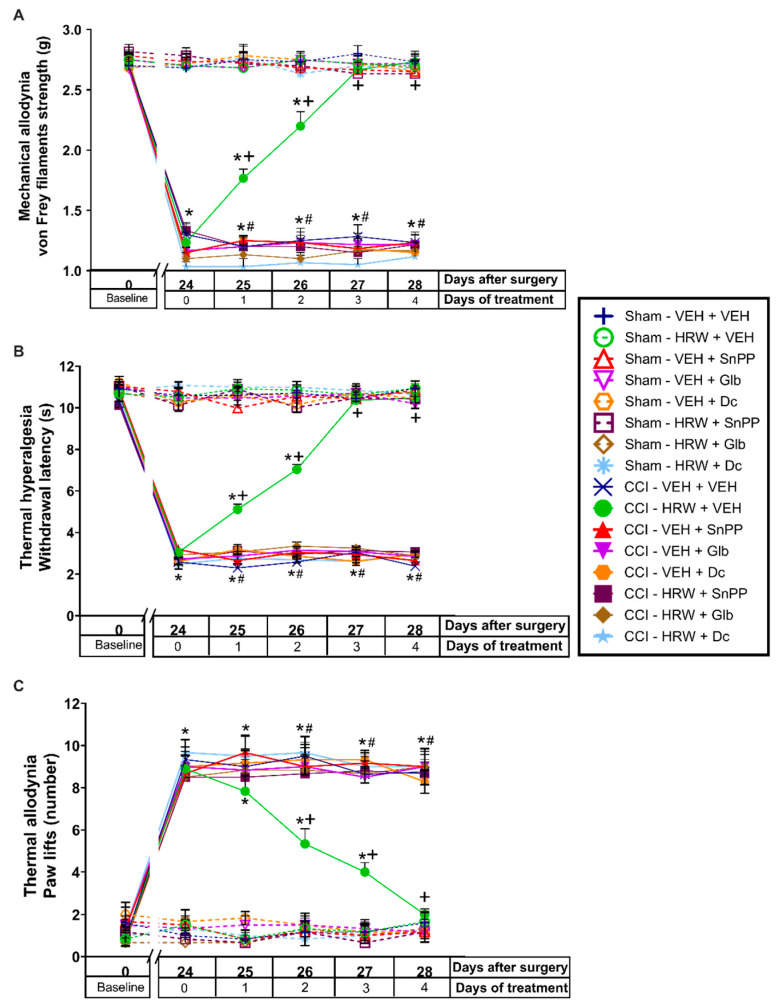
Reversion of the antinociceptive actions of HRW by the co-administration with HO-1 or NQO1 inhibitors and a K_ATP_ channels blocker during 4 consecutive days in the ipsilateral paws of CCI mice. The effects of the repetitive co-treatment of VEH or HRW with SnPP, dicoumarol (Dc) or glibenclamide (Glb) administered at 2T from days 25 to 28 after surgery, on the mechanical allodynia (**A**), thermal hyperalgesia (**B**) and thermal allodynia (**C**) induced by CCI are represented. Sham-operated mice are also displayed. In all figures, for each treatment and day analyzed, * indicates significant differences vs. their respective sham-operated treated animals, + vs. CCI-VEH-treated animals and # vs. CCI–HRW–VEH-treated animals (*p* < 0.05, one-way ANOVA followed by the Bonferroni test). Mean values ± SEM (n = 6 animals per group).

The three-way repeated measures ANOVA showed significant effects of surgery, treatment, and days of treatment (*p* < 0.001); and interactions between surgery and treatment (*p* < 0.001), surgery and days of treatment (*p* < 0.001), treatment and days of treatment (*p* < 0.001), and among the three factors (*p* < 0.001) in the mechanical allodynia (Figure 4A), thermal hyperalgesia (Figure 4B) and thermal allodynia (Figure 4C).

The results showed that the mechanical antiallodynic (Figure 4A) and thermal antihyperalgesic (Figure 4B) actions produced by HRW were reversed with its co-administration with SnPP, dicoumarol and glibenclamide, from day 1 to 4 of treatment (*p* < 0.001, one-way ANOVA vs. CCI-mice treated with HRW). While the thermal antiallodynic effects of HRW (Figure 4C) were also reversed with the co-administration with SnPP, dicoumarol and glibenclamide, but from day 2 to 4 of treatment (*p* < 0.001, one-way ANOVA vs. CCI-mice treated with HRW).

In all tests, treatment with SnPP, dicumarol, or glibenclamide injected alone did not change the nociceptive effects elicited by CCI (Figure 4A–C), and no effects of either treatment were observed in the contralateral paws of CCI- or sham-operated animals at none of the evaluated days (data not shown).

### 3.4. Treatment with CoPP Potentiated the Antinociceptive Effects of HRW

The possible interaction between HO-1 and H_2_ were studied by evaluating the antinociceptive effects produced by the co-treatment of CoPP with HRW, both injected at 2T during four consecutive days.

For the three evaluated nociceptive responses (mechanical allodynia, thermal hyperalgesia, and thermal allodynia) the three-way repeated measures ANOVA showed significant effects of the surgery, treatment, and days of treatment (*p* < 0.001); interactions between each two of them (*p* < 0.001), and among the three factors (*p* < 0.001).

**Figure 5:**
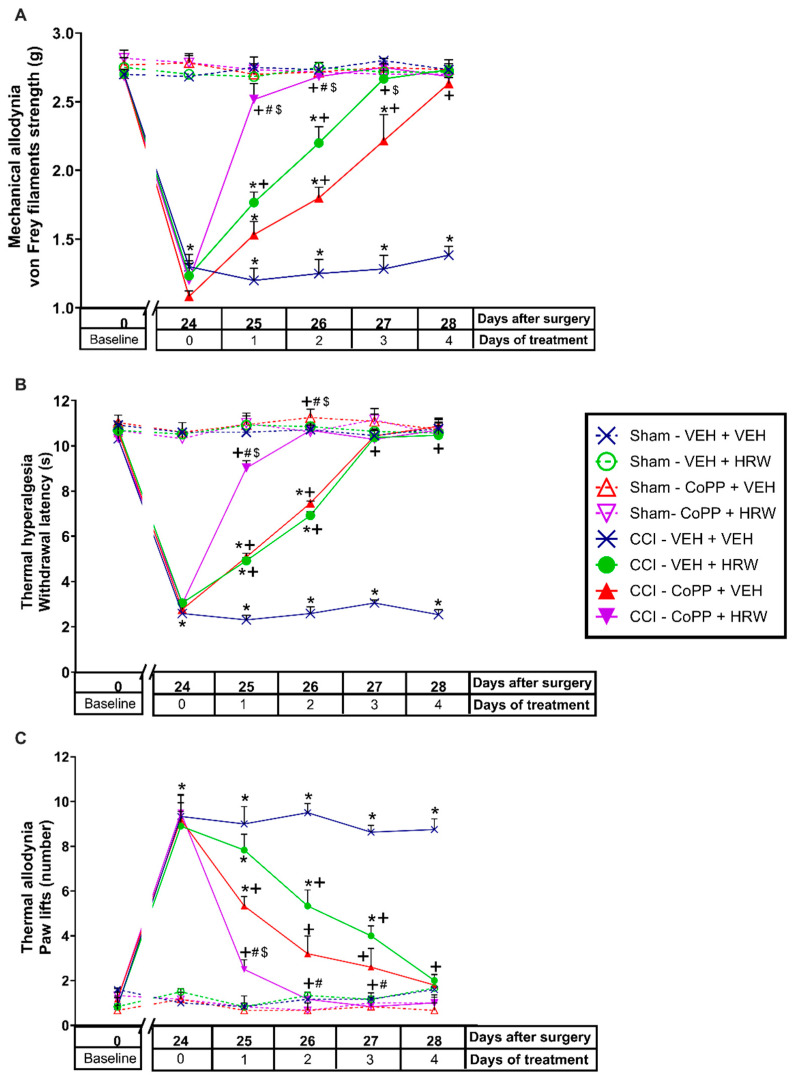
The co-administration of CoPP with HRW improved the analgesic properties of each of both treatments administered individually. The mechanical antiallodynic (**A**), thermal antihyperalgesic (**B**), and thermal antiallodynic effects (**C**) produced by the repetitive administration of CoPP, HRW and CoPP plus HRW at 2T from days 25 to 28 after surgery are represented. Sham-operated mice are also displayed. In all tests, for each treatment and day evaluated, * indicates significant differences vs. sham-operated VEH plus VEH treated animals, + vs. CCI-VEH plus VEH treated animals, # vs. CCI–VEH plus HRW treated animals and $ vs. CCI-CoPP plus VEH treated mice (*p* < 0.05, one-way ANOVA followed by the Bonferroni test). Mean values ± SEM (n = 6 animals per group).

The results showed that the decrease on the withdrawal threshold to von Frey filaments (*p* < 0.001, vs. sham-operated mice treated with VEH; Figure 5A), the withdrawal latency to thermal stimulus (*p* < 0.001, vs. sham-operated mice treated with VEH; Figure 5B) and the enhanced number of paw lifts to cold stimulus (*p* < 0.001 vs. sham-operated mice treated with VEH, Figure 5C) reflected in the ipsilateral paws of CCI-animals, from day 24 to 28 after surgery, were more rapidly inhibited with the co-administration of CoPP with HRW than by the administration of each of them administered separately. Next, while the single administration of HRW or CoPP completely inhibited the mechanical allodynia, thermal hyperalgesia, and thermal allodynia after 3 and 4 days of treatment (*p* < 0.05, one way ANOVA vs. CCI-mice treated with VEH plus VEH), the co-treatment of CoPP with HRW completely inhibited these nociceptive responses at the first day of treatment and their effects were higher than those produced by each treatment individually administered (*p* < 0.05 vs. CCI-VEH + HRW and CCI-CoPP + VEH treated mice).

For each test and treatment day, no differences between the antinociceptive effects produced by HRW or CoPP administered alone were observed. No effect of any treatment was detected in the ipsilateral paws of sham-operated mice (Figure 5), nor in the contralateral paws of CCI- or sham-operated mice in any of the three tests performed (data not shown).

### 3.5. The Effects of HRW Treatment on the Expression of the Antioxidant Enzymes HO-1, SOD-1 and NQO1 in the AMG and DRG of CCI-Mice

To assess the possible impact of treatment with HRW on the central and peripheral endogenous antioxidant pathway, the protein levels of HO-1, SOD-1 and NQO1 enzymes were analyzed in the AMG and DRG.

**Figure 6:**
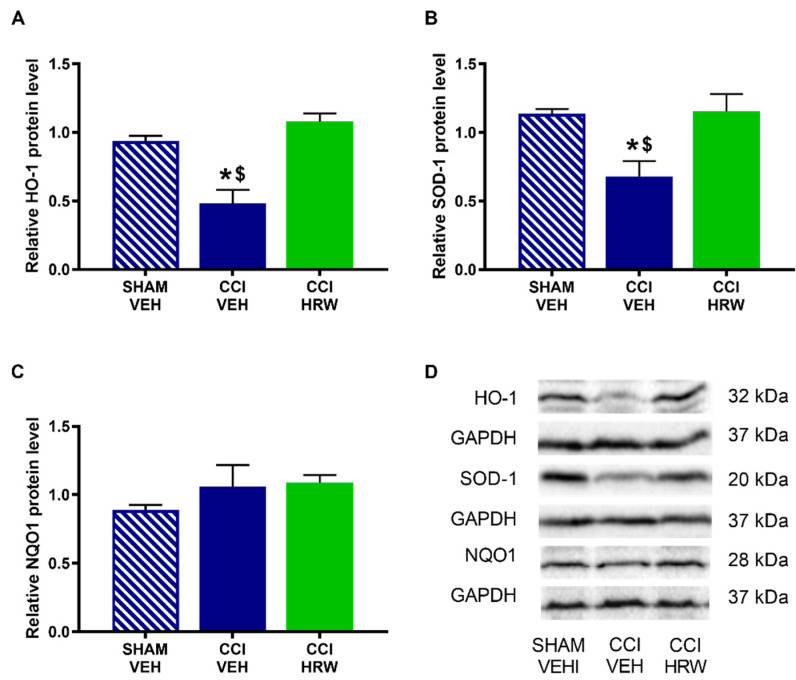
The effect of HRW treatment on the protein levels of HO-1, SOD-1 and NQO1 in the AMG of animals with CCI-induced neuropathic pain. The levels of HO-1 (**A**), SOD-1 (**B**) and NQO1 (**C**) of CCI-animals treated with VEH or HRW are represented. Sham-operated mice treated with VEH are also displayed. Proteins are expressed relative to GAPDH levels. Representative blots of HO-1, SOD-1 and NQO1 are shown (**D**). In all panels, * represents significant differences vs. sham-operated mice treated with VEH and $ vs. CCI-mice treated with HRW (*p* < 0.05, one-way ANOVA followed by the Bonferroni test). Mean values ± SEM (n = 3 samples per group).

In the AMG, a reduced expression of HO-1 (Figure 6A) and SOD-1 (Figure 6B) was observed in CCI-animals treated with VEH (*p* < 0.05, one-way ANOVA vs. sham-operated mice treated with VEH). In both cases, treatment with HRW avoided this decreased expression. No differences were observed on NQO1 levels in any of the analyzed groups (Figure 6C).

**Figure 7:**
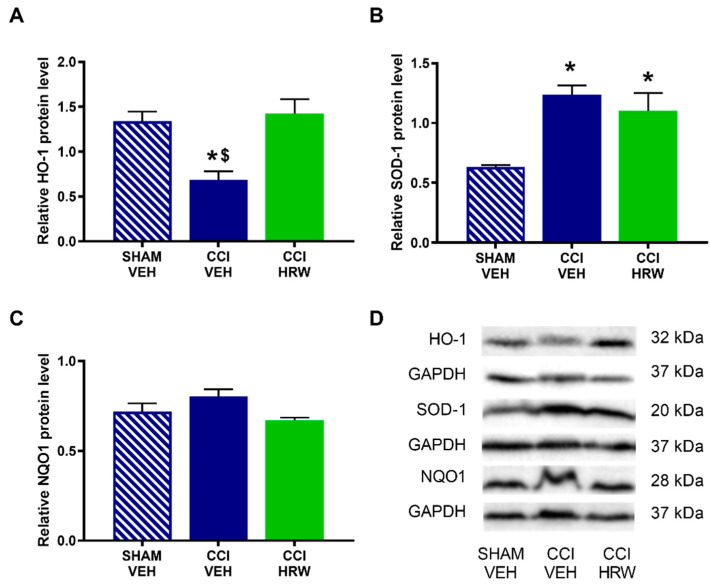
The effect of HRW treatment on the protein levels of HO-1, SOD-1 and NQO1 in the DRG of animals with neuropathic pain. The levels of HO-1 (**A**), SOD-1 (**B**) and NQO1 (**C**) of CCI-animals treated with VEH or HRW are represented. Sham-operated mice treated with VEH are also shown. Proteins are expressed relative to GAPDH levels Representative blots of HO-1, SOD-1 and NQO1 are shown (**D**). In all panels, * represents significant differences vs. sham-operated mice treated with VEH and $ vs. CCI-mice treated with HRW (*p* < 0.05, one-way ANOVA followed by the Bonferroni test). Mean values ± SEM (n = 3 samples per group).

In the DRG, there was also a reduction on the HO-1 expression in CCI-mice treated with VEH (*p* < 0.05, one-way ANOVA vs. sham-operated mice treated with VEH), which was reversed by HRW treatment (Figure 7A). In this tissue, the improved expression of SOD-1 observed in CCI-mice treated with VEH was retained after HRW treatment (*p* < 0.05, one-way ANOVA vs. sham-operated mice treated with VEH, Figure 7B). Finally, as in the AMG, no differences in the NQO1 levels were observed among groups (Figure 7C).

### 3.6. The Impact of HRW Treatment on the Expression of p-IKBα, p-AKT, BAX and 4-HNE in the AMG and DRG of CCI-Mice

Different pathways that might be modulated by HRW treatment in CCI-mice were explored. Thus, we evaluated the expression of p-IKBα as a indicator of the inflammatory response through the NF-κB pathway, p-AKT as related to the nociceptive response, BAX as a cellular death marker and 4-HNE as related to oxidative stress.

**Figure 8:**
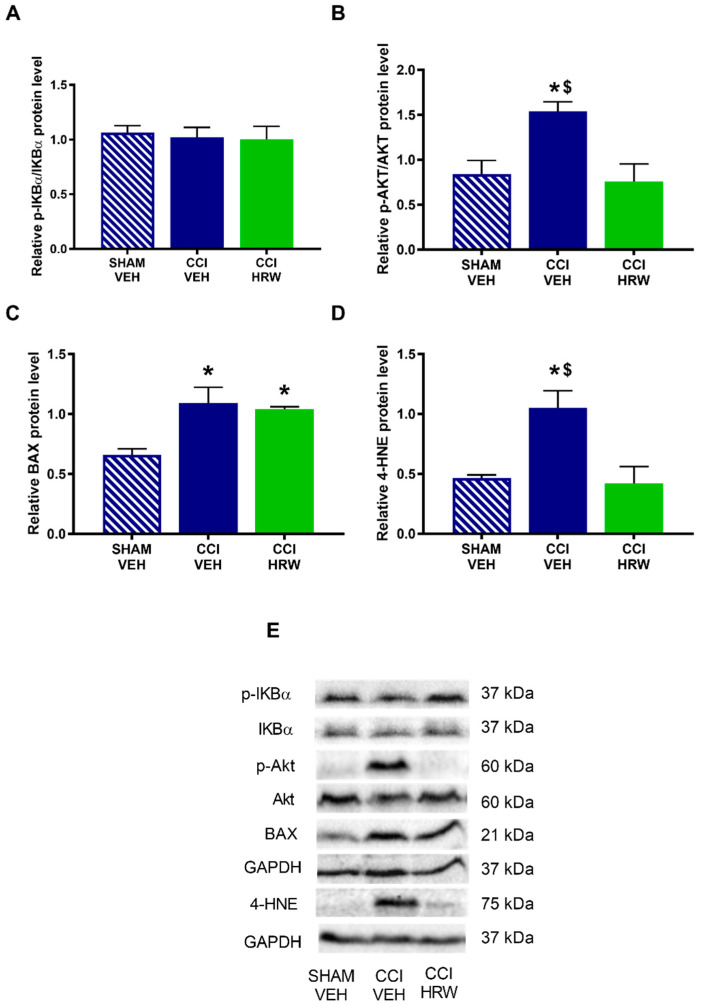
The effect of HRW treatment on the protein levels of p-IKBα, p-AKT, BAX and 4-HNE in the AMG of animals with neuropathic pain. Levels of p-IKBα (**A**), p-AKT (**B**), BAX (**C**) and 4-HNE (**D**) of CCI-mice treated with VEH or HRW are displayed. Sham-operated mice treated with VEH are also shown. p-IKBα and p-AKT are expressed relative to their corresponding total protein levels whereas BAX and 4-HNE are expressed relative to GAPDH levels. Representative blots of p-IKBα, IKBα, p-AKT, AKT, BAX and 4-HNE are shown (**E**). In all panels, * represents significant differences vs. sham-operated mice treated with VEH and $ vs. CCI-mice treated with HRW (*p* < 0.05, one-way ANOVA followed by the Bonferroni test). Mean values ± SEM (n = 3 samples per group).

The results showed that while no differences on the expression p-IKBα (Figure 8A) were observed, a significant increase in the p-AKT (Figure 8B), BAX (Figure 8C) and 4-HNE (Figure 8D) was detected in the AMG of CCI-mice treated with VEH (*p* < 0.05, one-way ANOVA vs. sham-operated mice treated with VEH). Treatment with HRW normalized the overexpression of p-AKT and 4-HNE, but not those of BAX.

**Figure 9:**
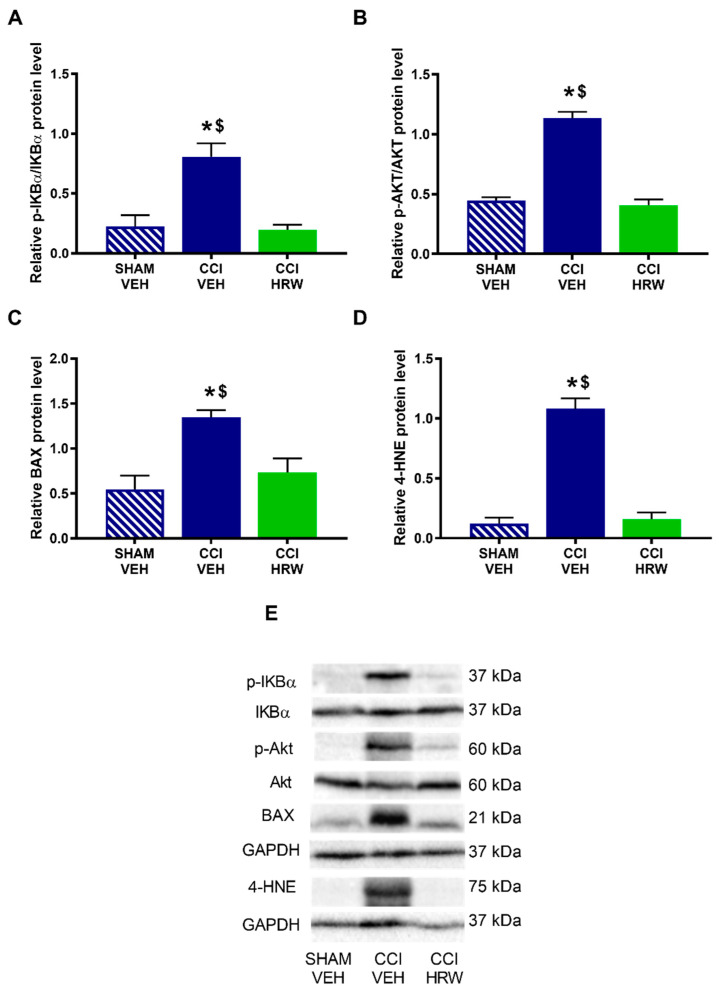
The effect of HRW treatment on the protein levels of p-IKBα, p-AKT, BAX and 4-HNE in the DRG of animals with neuropathic pain. The expression of p-IKBα (**A**), p-AKT (**B)**, BAX (**C**) and 4-HNE (**D**) in CCI-mice treated with VEH and HRW are displayed. Sham-operated mice treated with VEH are also shown. p-IKBα and p-AKT are expressed relative to their corresponding total protein levels whereas BAX and 4-HNE are expressed relative to GAPDH levels. Representative blots of p-IKBα, IKBα, p-AKT, AKT, BAX and 4-HNE are shown (**E**). In all panels, * represents significant differences vs. sham-operated mice treated with VEH and $ vs. CCI-mice treated with HRW (*p* < 0.05, one-way ANOVA followed by the Bonferroni test). Mean values ± SEM (n = 3 samples per group).

In the DRG, nerve injury increased the expression of p-IKBα (Figure 9A), p-AKT (Figure 9B), BAX (Figure 9C) and 4-HNE (Figure 9D) (*p* < 0.05, one-way ANOVA vs. sham-operated mice treated with VEH) and all of them were inhibited with HRW treatment.

## 4. Discussion

This study showed that the systemic therapeutic treatment with HRW inhibited the mechanical allodynia, thermal hyperalgesia and thermal allodynia induced by CCI and the emotional deficits associated in mice. These data also revealed the involvement of the endogenous antioxidant system and the K_ATP_ channels on the analgesic effects induced by HRW treatment. Moreover, the co-administration of CoPP with HRW improved the painkiller actions of both treatments suggesting a positive interaction between them. The results also showed the regulatory actions of treatment with HRW in the nociceptive, inflammatory, apoptotic and/or oxidative outcomes generated by CCI in the AMG and/or DRG of animals with neuropathic pain.

Previous studies have shown the protective effects of H_2_, as a prophylactic therapy, in reducing oxidative stress, inflammation, and apoptosis in rodents [11,24,27]. Nevertheless, the potential use of H_2_ as a therapeutic strategy for neuropathic pain has been poorly investigated. This study demonstrated that treatment with HRW, administered at 1T or 2T from days 22 or 25 after nerve injury, completely inhibited the nociceptive deficits induced by CCI in mice afterwards 7 or 4 consecutive days of treatment, respectively. Thus, revealing the curative potential of the intraperitoneal administration of HRW during neuropathic pain. Our data also demonstrated that the two daily administrations of HRW were more effective than one per day in reducing nociception. In compliance with these findings, the oral and intrathecal prophylactic treatment with HRW or HRS also reduced neuropathic pain produced by the partial or total sciatic nerve ligation in rats [24,25,27]. Thus, revealing the potential use of HRW for treating neuropathy not only as a prophylactic strategy but also as a novel therapeutic approach.

Concerning the emotional disorders, some studies have shown the promising use of H_2_ for treating the depressive-like behaviors provoked by mild chronic unpredictable stress [29] and the anxiety-like behaviors associated with morphine-withdrawal [34] or with a subarachnoid hemorrhage in rodents [35]. Despite that, the effects of H_2_ on the emotional disorders accompanying neuropathic pain have not been studied. This study revealed that treatment with HRW administered at 1T for 7 consecutive days or at 2T for 4 consecutive days both inhibited the anxiety- and depressive-like behaviors accompanying CNP. Therefore, and considering the greater effectiveness of HRW administered at 2T versus at 1T in inhibiting neuropathic pain, this study proposed that the curative treatment with HRW administered at 2T might be a good alternative for the management of CNP and the related anxiety- and depressive-like behaviors in mice. In agreement with this proposal, other studies have also validated the use of several antioxidants or slow-releasing H_2_S donors for treating neuropathic pain and its related comorbidities in mice [17,36].

The most relevant characteristic of H_2_ treatment in several disorders such as ischemia/reperfusion injury or Alzheimer’s disease is its antioxidative properties [20]. Next, we evaluated if the activation of the endogenous antioxidant system and/or the K_ATP_ channels are two possible paths involved in the analgesic actions of HRW under neuropathic pain conditions. We assessed the effects of HRW, intraperitoneally administered at 2T during four consecutive days, in animals co-treated with SnPP (HO-1 inhibitor), dicoumarol (NQO1 inhibitor) or glibenclamide (K_ATP_ channels blocker). The fact that all of them reversed the antiallodynic and antihyperalgesic effects of HRW revealed the participation of the antioxidant enzymes (HO-1 and NQO1) and the K_ATP_ channels in the analgesic actions of HRW during neuropathic pain. These data agreed with other findings showing the reversion of the analgesic effects induced by HRS with SnPP in rats with neuropathic pain [11] as well as with the contribution of the NQO1 enzyme and K_ATP_ channels in the pain reliever effects of H_2_S donors in animals with chronic joint or inflammatory pain [14,31].

Tacking account these results, the possible enhancement of the analgesic actions of HRW treatment with its co-administration with the HO-1 inducer, CoPP was furthermore investigated. Data showed that the co-administration of CoPP with HRW produced a greater analgesic effect than that made by both treatments administered separately and that only one day of treatment was enough to fully inhibit the nociceptive replies caused by CCI, as compared with the 3 or 4 days of treatment required by CoPP or HRW administered alone. Thus, revealing a positive interaction between HO-1 and H_2_ systems in inhibiting CNP. In line with these data, a positive interaction between HO-1/CO with other gases, for example H_2_S or nitric oxide, has been also proved in other pain models [13,14]. Our data suggested the combination of CoPP with HRW as a good antinociceptive strategy for CNP.

The cooperative interaction among HRW and HO-1 was further supported by the increased expression of HO-1 induced by HRS in neuropathy [11,24] and other pathologies [20]. In accordance with that, this study showed that the downregulation of HO-1 provoked by CCI in the DRG was stabilized with HRW treatment. These results together with (i) the reversal of the analgesic effects of HRW with SnPP or dicumarol, (ii) the potentiation of the analgesic actions of HRW with the co-treatment with CoPP and (iii) the maintenance of high levels of SOD-1 in the DRG of HRW-treated mice, evidenced the importance of the endogenous antioxidant system in the analgesic actions of HRW during CNP. These results might justify the enhanced painkiller actions produced by the co-administration of CoPP with HRW. Furthermore, the normalization of the oxidative stress (4-HNE), cell death (BAX), nociceptive (p-AKT) and inflammatory responses (p-IKBα) incited by CCI after HRW treatment in the DRG revealed the protective role of H_2_ in CNP and suggested that the protective actions of this molecule might be also linked with its pain-relieving properties. In line with our data, earlier findings also proved the key role performed by HO-1, NQO1 and/or SOD-1 enzymes in the antinociceptive actions produced by some H_2_S and HO-1 activators in neuropathic pain [37,38].

The AMG is one of the brain regions most implicated in regulating the anxiety- and depressive-like behaviors, and this role has been also demonstrated in different rodent models of CNP [3]. Nevertheless, the potential modulatory effects induced by the systemic administration of HRW in this brain area have not yet been evaluated. Our results demonstrated that treatment with HRW also stabilized the down regulation of HO-1 and the increased expression of 4-HNE and p-AKT in the AMG, as it happens in the DRG. Moreover, the downregulation of SOD-1 generated by CCI was also normalized with HRW in this brain area, thus revealing the central antioxidant properties of this treatment in mice with neuropathic pain. Therefore, and taking into consideration the anxiolytic and antidepressant effects produced by several antioxidant compounds in animals with neuropathic pain [36,39], our data suggested that the antioxidant activities induced by HRW treatment in the AMG might be implicated in its management of the emotional disorders associated with CNP. These findings were supported by the contribution of the antioxidant system in the anxiolytic and antidepressant outcomes induced by diallyl disulfide and GYY4137, two slow-releasing H_2_S donors, in mice with CNP [18]. Nevertheless, more experiments are needed to confirm this hypothesis.

This study has some limitations, such as that only evoked pain was evaluated, and it was only performed on male mice.

## 5. Conclusions

In conclusion, this study showed: (i) the potential therapeutic use of HRW for treating nerve injury-induced CNP and the associated emotional deficits, (ii) the activation of the endogenous antioxidant system and K_ATP_ channels as possible mechanisms of action of HRW in inhibiting neuropathic pain, (iii) a positive interaction between HO-1 and H_2_ systems in modulating nerve-injury induced neuropathy and (iv) the antioxidant, antinociceptive, anti-inflammatory and/or antiapoptotic actions of treatment with HRW in the DRG and/or AMG of mice with CNP.

These data suggest that treatment with HRW might be an interesting therapeutic strategy for nerve-injury induced CNP and its associated mood disorders.

## Data Availability

Data is contained within the article.

## Abbreviations

1T	1 time/day
2T	2 times/day
4-HNE	4-hydroxy-2-nonenal
AMG	amygdala
ANOVA	analysis of variance
BAX	Bcl2-associated X
CCI	chronic constriction of the sciatic nerve
CNP	chronic neuropathic pain
CNS	central nervous system
CO	carbon monoxide
CoPP	cobalt protoporphyrin IX
DMSO	dimethyl sulfoxide
DRG	dorsal root ganglia
EPM	elevated plus maze
FST	forced swimming test
H_2_	molecular hydrogen
H_2_S	hydrogen sulfide
GAPDH	glyceraldehyde-3-phosphate dehydrogenase
HO-1	heme oxygenase 1
HRS	hydrogen-rich saline
HRW	hydrogen-rich water
K_ATP_	ATP sensitive potassium
NF-κB	nuclear factor-κB
NQO1	NAD(P)H: quinone oxidoreductase 1
Nrf2	nuclear erythroid factor 2-related factor 2
p-Akt	phosphorylated protein kinase B
PBST	phosphate-buffered saline plus Tween 20
p-IKBα	phosphorylated-NF-κB inhibitor alpha
PI3K	phosphoinositide 3-kinase
PNS	peripheral nervous system
ROS	reactive oxygen species
SEM	standard error of the mean
SnPP	tin protoporphyrin IX
SOD-1	superoxide dismutase 1
TBST	Tris-buffered saline plus Tween 20
TST	tail suspension test
VEH	vehicle
